# How to polarise an interface with ions: the discrete Helmholtz model†

**DOI:** 10.1039/d0sc00685h

**Published:** 2020-05-18

**Authors:** Grégoire C. Gschwend, Astrid Olaya, Hubert H. Girault

**Affiliations:** Laboratoire d’Electrochimie Physique et Analytique (LEPA), École Polytechnique Fédérale de Lausanne (EPFL) Rue de l’Industrie 17 CH-1951 Sion Switzerland hubert.girault@epfl.ch

## Abstract

The distribution of electrolytes in an electric field usually relies on theories based on the Poisson–Boltzmann formalism. These models predict that, in the case of a metallic electrode, ionic charges screen the electrode potential, leading to concentration-dependent ion distributions. This theoretical framework was first applied at solid–liquid interfaces and then transposed to soft interfaces. However, in this latter case, the potential in which the electrolytes evolve is not homogeneous, which is less amenable to a mean-field description. In this report, we show that at polarised soft interfaces the potential difference takes place between two closely interacting ionic monolayers. In this configuration, ions of opposite charges directly neutralise each other leading to an absence of diffuse layers and charge screening by surrounding ions. Thus, independently of the electrolyte concentrations, the surface charge density is a linear function of the potential difference, which results in a constant capacitance.

## Abstract 2

The discrete nature of the charge carriers at polarised soft interfaces yields sharp potential profiles as well as concentration and potential independent capacitances.

## Graphical abstract

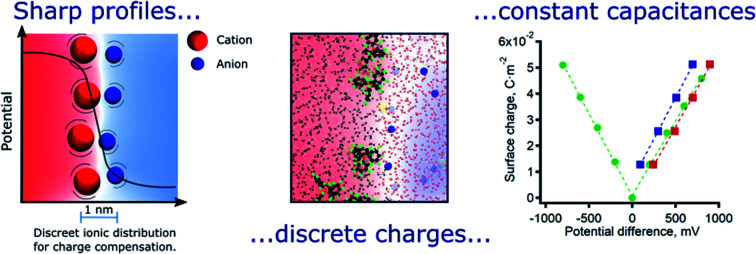

## Introduction

The behaviour of charged particles at electrified interfaces is important in many domains of science, from plasma physics to chemistry and biology. In electrochemistry, the Gouy–Chapman model was one of the first theories that described the potential and charge concentration profiles near a polarised electrode. This classical model is actually a solution of the more general Poisson–Boltzmann equation, solved assuming a planar symmetry of the electrode. Later, Debye and Hückel used the same framework and derived a model to calculate the activity coefficient of electrolytes in dilute solutions. This model assumes that the ionic atmosphere around an ion in solution follows the Poisson–Boltzmann equation, but in a spherical symmetry. However, already in 1933, Onsager criticised the way this theory used the Poisson–Boltzmann equation and showed that only under well-defined circumstances could the potential of average force be replaced by the electrostatic potential of the central ion.^1^ His concerns were nevertheless neglected in applications to solid–liquid interfaces as, in the Gouy–Chapman–Stern theory, a large fraction of the potential difference drops between the electrode and specifically adsorbed ions, implying therefore that the diffuse layer experiences only a reduced potential, far from the electrode, which allows the approximations of the Gouy–Chapman theory to remain valid.^2,3^ Nevertheless, this “buffering action of the condensed phase”, as described by Fixman,^2^ works because the ions of the Stern layer only partially cancel the homogeneous surface charge of the electrode. Nevertheless, despite some reservations, the Gouy–Chapman model was transposed to liquid–liquid interfaces, as a way to describe electrolytes distributions close to these surfaces.^4^ This theory is now a cornerstone of the electrostatics of cell membranes.^5^ However, an important difference compared to the solid–liquid interface is that, in the case of soft interfaces, the ionic species carry the polarisation of both phases. Thus, these interfaces no longer fulfil the assumption that the electrolytes are in a homogeneous electric field. As a consequence, the Gouy–Chapman model often fails to explain the results of electrochemical experiments at these interfaces, such as capacitance curves^6^ or surface tension measurements.^7^

In order to improve upon the Gouy–Chapman model, Schlossman *et al.* included ion–solvent^8^ and then ion–ion^9^ correlations in the Poisson–Boltzmann equation, hence solving part of the concerns raised by Onsager. With these improvements, the so-called “Poisson–Boltzmann Potential of Mean Force” model (PB-PMF) could simulate X-ray reflectivity at the polarised liquid–liquid interface, in a remarkable concordance with the experimental results. Nevertheless, this model still relied on an approach where particles interactions are considered on average in an effective potential.

One of the difficulties in the study of soft interfaces is the limited number of experimental methods available to probe their structure. Thus, the work of Schlossman *et al.* constituted an interesting approach, although X-ray reflectivity only probes the electron density and depends on a model to provide structural details of ionic layers. In this respect, second-order optical spectroscopy is a valuable tool to observe the structure of the polarised soft interfaces because it is inherently surface sensitive and specific to molecular orientation^10^ and electric fields.^11^ Recently, using sum frequency generation, Dreier *et al.* have shown that water reorientation and counterions permeation contributed to the reduction of the surface potential of soft interfaces,^12^ questioning therefore the relevance of mean-field models.

In this report, we present qualitative features of the structure of the double layer of the polarised water–dichloroethane (DCE) interface that were not predicted by models based on the Poisson–Boltzmann formalism. We support our conclusions with molecular dynamics simulations, surface second harmonic generation (SHG) and electrochemical impedance spectroscopy (EIS). Our results show that, independently of the electrolyte bulk concentrations, the surface charge density at the interface between the aqueous and organic phases depends linearly on the Galvani potential difference between the two phases. This observation implies therefore that the capacitance of this interface is constant. Furthermore, we found that the interface is devoid of diffuse layers since the potential difference drops in its totality between two sharp ionic layers. We explain this marked difference from the solid–liquid interface by the fact that the polarisation of the interface is supported by ions interacting at short distances (typically less than 5 Å), in localised but mobile potentials, which allows a direct compensation of their charges and prevent the charge screening mechanisms.

## Results and discussion

**Fig. 1:**
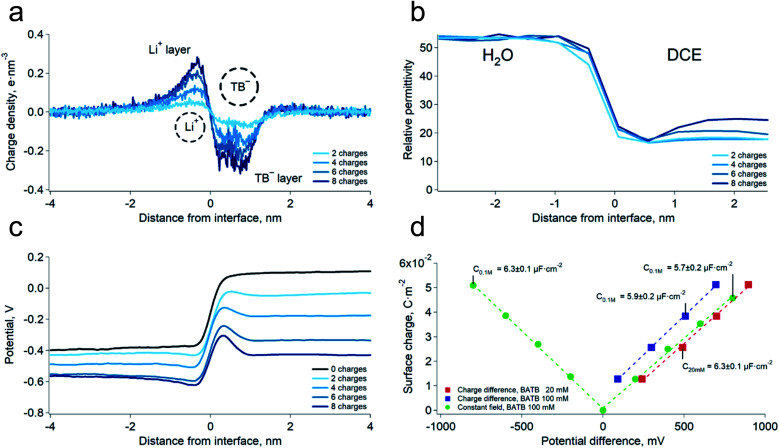
(a) Simulated charge density profiles at the water–DCE interface at various potential differences. LiCl 200 mM, BATB 100 mM, polarisation induced by the “charge difference” method (see Experimental part). The dashed circles represents the Li^+^ hydrated diameter and TB^−^ diameter. (b) Relative permittivity profiles of the interface at various potential differences. Bulk values are those expected from the molecular models. The permittivity was calculated in slices of 0.5 nm. (c) Simulated potential profiles at the water–DCE interface (LiCl 200 mM, BATB 100 mM). (d) Surface charge densities as a function of the interfacial potential difference at two BATB concentrations and with different polarisation methods. The corresponding value of the capacitance is, on average, 6.1 ± 0.3 μF cm^−2^.

We first carried out two series of molecular dynamics simulations of the polarised water–DCE interface. The electrolyte concentration was 200 mM lithium chloride (LiCl) in the aqueous phase and 100 mM bis-triphenyl phosphoranylidene ammonium tetrakis-pentafluoro phenyl borate (BATB) in the DCE phase and the simulations boxes were 5 × 5 × 45 nm wide, with an equal volume for each phase (see Experimental part). In the first series, we imposed the polarisation of the interface by creating an excess of charges in one phase, compensated by the same excess (of opposite sign) in the other phase. In the second series, we polarised the interface by applying a constant electric field through the simulation box. In this case, we chose the magnitude of the electric field to be such that the potential difference throughout the box was the desired one.^13^ In both cases, we observed the formation of two sharp ionic layers at the interface and no diffuse layers (Fig. 1a). These results are similar to those of Luo *et al.* who used the PB-PMF model.^8^ However, in our simulations, the charge profiles are antisymmetric, as opposed to those predicted by usual double layer models.^14^ Indeed, these usually assume that the permittivity at the interface is that of each bulk phases which, once translated into boundary conditions on the electric field (*ε*_w_*E*(0^+^) = *ε*_o_*E*(0^−^) at the interface, located at origin), implies a discontinuity of the charge distribution, because the charge is the first derivative of the electric field and is therefore constrained by the ratio of the dielectric constants. However, our simulations show that the permittivity of the aqueous phase decreases at approximately 1 nm from the interface, reaching that of the organic phase (Fig. 1b). This observation is consistent with the preferred orientation of the water molecules at liquid–liquid interfaces,^10^ which according to the Kirkwood–Fröhlich equation, decreases the variance of total dipole moment and therefore the permittivity.^15^ Thus, the simulations show that the ions at the aqueous side of the interface are in a medium of reduced permittivity, which favours electrostatic interactions. This observation leads us to think that there is a significant ion–ion correlation through the interface, contrary to what had been previously suggested by Schlossman *et al.* who assumed a large permittivity of the aqueous side of the interface^14^ or worked with nitrobenzene, whose permittivity is much larger than that of DCE.^16^ Nevertheless, no stable ion association was observed in the simulations.

**Fig. 2:**
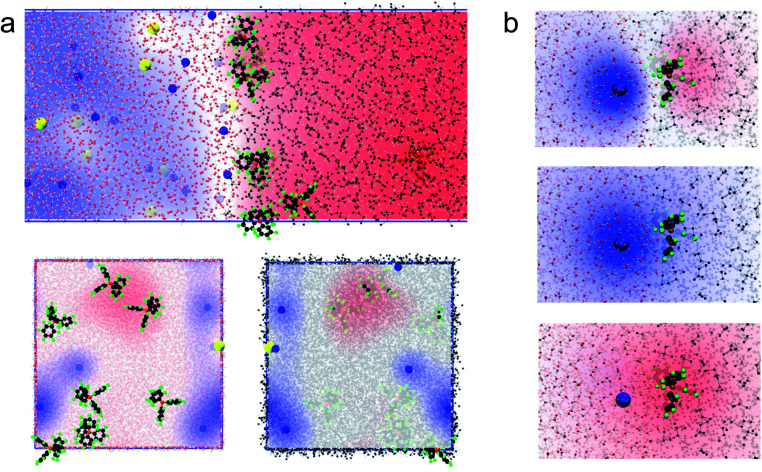
(a) Simulated electrostatic potential map between the ion layers at the water–DCE interface. Side view (top), view from the organic phase (bottom left) and from the aqueous phase (bottom right). Compared to a solid–liquid interface, the potential sensed by the electrolytes is inhomogeneous. Li^+^ is blue, carbon black, fluoride green, chloride yellow, oxygen red, hydrogen white and boron orange. The blue regions have a positive potential while the red regions have a negative potential. (b) Electrostatic potential of closely interacting Li^+^ and TB^−^ ions: total potential (top), Li^+^ alone (middle) and TB^−^ alone (bottom) at the water–DCE interface. The potential of the lithium cation cancels that of the TB^−^ anion. The contribution from the solvents has been removed for clarity.

We then computed the electric potential profiles from the simulations by double integration of the charge densities of the solvents and ions (Fig. 1c and S3†). We observed that the potential dropped sharply at the interface, over slightly more than 1 nm (this analysis is however complicated by the potential of the oriented water dipoles at the interface, a well-known artefact of molecular dynamics simulations^17^). Nevertheless, the sharp potential profiles explain the absence of a diffuse layer since the totality of the potential difference drops between the ions. Thus, ions located behind the interfacial layers do not sense any potential. Soft interfaces make possible such a direct compensation of the interfacial charges because the polarisation of both phases is carried by point charges. Indeed, the ions in each layer sense an inhomogeneous potential (Fig. 2a) as opposed to the solid–liquid interface, where the surface charge of the electrode is distributed over its whole surface while the charges of the electrolytes are localised. Thus, the ions never spatially compensate the totality of the electrode surface charge, which explains the presence of a diffuse layer, and justifies the mean-field approach of the classical models. Furthermore, in this case, all the ions in solution sense the electrode potential, which explains the dependence of the diffuse layer on the electrolyte concentration and therefore the screening effect. On the other hand, we observed a one-to-one interaction between the ion layers at the water–DCE interface, which allows a complete compensation of their charges and makes them less sensitive to surrounding ions. Therefore, no charge screening effect is observed. This is illustrate in Fig. 2b, where we show the cancellation of the electrostatic potentials of two closely interacting ions.

In order to verify the absence of screening effect, we carried further simulations at lower organic phase supporting electrolyte concentrations, *i.e.* 20 mM. As expected, we observed that the surface charge density was nearly independent on the bulk electrolyte concentration (Fig. 1d and S3†). Furthermore, we found that the surface charge was a linear function of the potential difference. This observation implies that the differential capacitance of the water–DCE interface, defined as d*σ*/d*φ* (where *σ* is the surface charge and *φ* the potential difference), is constant and, in the simulations, equal to 6.1 ± 0.3 μF cm^−2^ on average. This observation is in marked opposition with the models of the double layer that predict a potential and concentration dependent capacitance.^18^

**Fig. 3:**
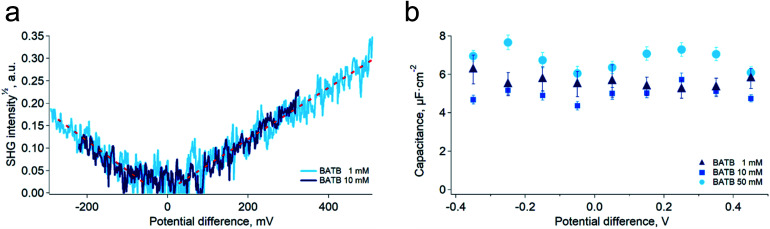
(a) Square root of the non-resonant (1000 nm–500 nm) second harmonic signal generated at the polarised water–DCE interface for two organic phase supporting electrolytes concentrations, 1 mM and 10 mM. Aqueous phase supporting electrolyte: LiCl 10 mM. The signal is proportional to the surface concentration of TB^−^ or BA^+^ ions. The surface charge density is independent on the bulk electrolyte concentrations. (b) Capacitance of the polarised water–DCE microinterface measured by electrochemical impedance spectroscopy in a two-electrode configuration. The capacitance does not depend on the potential difference nor on the concentration. Aqueous supporting electrolyte: LiCl 10 mM, organic phase supporting electrolyte indicated in the legend.

As a mean to support the hypothesis of a constant capacitance observed in the simulations, we carried out surface second harmonic generation measurements of the polarised water–DCE interface (see Experimental part and Fig. S4†). In these experiments, the second harmonic signal originates from the aromatic rings of the organic phase supporting electrolytes, BA^+^ and TB^−^,^19,20^ and provides therefore a direct experimental way to measure the ion concentration at the organic side of the interface, since the signal intensity is proportional to the square of the concentration. Here, the aqueous phase was a 10 mM LiCl solution, while the organic phase was a 1 mM or 10 mM BATB solution in DCE. In agreement with our simulations, we found that the surface concentration of BA^+^ and TB^−^ depends linearly on the potential difference and is independent on the electrolyte bulk concentration (Fig. 3a). Interestingly, Conboy & Richmond already reported similar results^20^ but analysed them in the framework of the modified Verwey–Niessen model. This model is theoretically close to the Gouy–Chapman theory and predicts that the surface charge is proportional to sinh(*fφ*), where *f* is the fraction of the potential difference, *φ*, that drops in the organic phase. In their study, they found a value of 0.1 for *f*, which is particularly small given the low permittivity of the organic phase compared to that of the aqueous phase. In light of our results, we think that such a low value actually supports the linear dependence on *φ*, because the argument of the hyperbolic sine is small, which implies that it could be approximated to the first order, *i.e.* as a linear function. It is important to note that the BATB concentrations used in the SHG experiments, 1 mM and 10 mM, are smaller than those used in molecular dynamics simulations, 20 mM and 100 mM. However, since we experimentally observe a linear dependence of the surface concentration already at 1 mM, and that experiments at 10 mM confirm this trend, we do not expect it to be different at higher concentrations.

Building on the present simulations and spectroscopic results, we carried out further experiments of electrochemical impedance spectroscopy at the water–DCE interface, in order to measure directly the capacitance of the interface. Here, we used a microhole-supported liquid–liquid interface in order to work in a two-electrode configuration.^21^ Indeed, several authors have reported that working with a four-electrode configuration creates artefacts at high-frequencies, mainly because of the stray capacitance induced by the organic phase reference electrode.^22–24^ The present setup consisted in two 2 mL Teflon compartments separated by a 30 μM thick Kapton film in which a hole of approximately 23 μM in diameter had been laser ablated (Experimental part and Fig. S5†). Two AgCl-coated silver wires served both as reference and as counter-electrodes. With this setup and the fitting model we used, the capacitance measured was nearly constant over the 800 mV wide potential window and did not clearly depend on the organic phase electrolyte concentration in the range 1 mM to 50 mM (Fig. 3b). Furthermore, the average value found experimentally, 5.8 ± 0.9 μF cm^−2^, was in good concordance with that of the simulations, 6.1 ± 0.3 μF cm^−2^. These results are markedly different from those observed in a four-electrode configuration and at lower frequencies, where the reported capacitance depends on the potential difference and electrolyte concentrations.^6,25,26^ We think however that at low frequencies, ion transfer, electric field induced convections and capillary waves at the interface are responsible for an artificially large capacitance. Indeed, this was suggested by Samec and co-workers who observed discrepancies between surface charge densities measured by electrocapillary and by capacitance measurements. They thus proposed that the later were artificially larger because of a wrong representation of the electrical properties of the interface by the classical circuit elements used to fit the impedance spectra, particularly because of the coupling between capillary waves and potential variations.^7,24,27–31^

The results obtained by impedance spectroscopy presented in this report with the organic salt BATB could be reproduced with a BATCPB (where TCPB stands for tetrakis(4-chlorophenyl) borate), a different organic phase supporting electrolyte (see Fig. S9 and S10†). This supports that our conclusions are not limited to a particular electrolyte. Furthermore, the constant capacitance of the ITIES and the linear dependence of the surface charge density on the polarisation imply a quadratic dependence of the electrocapillary curves. Our results thus provide an explanation for the better agreement of the electrocapillary curves with quadratic fittings than with hyperbolic cosine that are predicted by the Gouy–Chapman model.^24,32^

## Conclusions

Overall, the work presented in this report provides evidence that models based on the Poisson–Boltzmann equation do not satisfactorily describe ion distributions at polarised liquid–liquid interfaces. If their limitations had already been discussed,^16,33^ they were still believed to provide qualitative predictions of the differential capacitance in terms of electrolyte concentrations and potential difference. In these models, the increase of the capacitance with the electrolyte concentration or with the interface polarisation were understood as originating from the screening of the surface potential by the free charges in solution. Here, however, we showed that the direct and localised compensation of the charges at the interface, implied by a close interaction of the ions in the double layer, makes them nearly insensitive to the presence of the other ions. Our results show the importance of discrete electrostatic interactions at soft interfaces, while such interactions are also known to be relevant at the solid–electrolyte interfaces.^34,35^

## Experimental

### Chemicals

Anhydrous lithium chloride (LiCl, >99%) was purchased from Sigma. Dichloroethane 99.5% (for electronic use) was obtained from Acros. Lithium tetrakis(pentafluorophenyl) borate ethyl etherate (LiTB) was purchased from Boulder Scientific, potassium tetrakis(4-chlorophenyl) borate (KTCPB, 98%) was obtained from Fluka. Bis(triphenylphosphoranylidene) ammonium chloride (BACl, 98%) was purchased from Fluka. All chemicals were used as received.

### Synthesis of BATB

Bis(triphenyl phosphoranylidene) ammonium tetrakis(penta fluorophenyl) borate (BATB) was synthesised by metathesis of LiTB and BACl as follows. A solution of 1.4 g of LiTB dissolved in 30 mL of a 30% ethanol and a solution of 920 mg of BACl in 30 mL of 30% ethanol where prepared. Then, the solution of BACl was slowly added to the solution of LiTB, under stirring. The precipitation of BATB is immediate. The thus-obtained solution was left under stirring for ten minutes and filtered with a Buchner funnel. The organic salt was then dried one hour in an oven at 80 °C. Finally, BATB was purified by recrystallization as follows. Dry BATB is dissolved in a minimum amount of acetone. Then, water is added drop-wise under stirring to the acetone solution until complete precipitation of BATB. The salt is then recovered by filtration with a Buchner funnel and washed with water. This procedure was repeated two times. We used the same protocol to synthesise BATCPB.

### Computational details

The molecular dynamics simulations were all carried out using GROMACS 2018.1.^36–42^

#### Topologies

The simulations contain six types of chemical species: water, dichloroethane (DCE), BA^+^, TB^−^, Li^+^ and Cl^−^. The water was simulated with the TIP4P model while the TFT molecules used the standard OPLS force field parameters. Li^+^ and Cl^−^ were simulated with the parameters of Jensen & Jorgensen.^43^ The geometries and partial charges of the organic ions were calculated from DFT simulations using GAMESS-US 2018.^44^ The exchange and correlation functional was the ω-B97XD while the basis set was a triple zeta plus polarisation of Ahlrichs *et al.*^45^ for both the geometries and charges. In the calculation of the partial charges, a finer Lebedev grid containing 120 radial points and 770 angular points was used. The charges were obtained by fitting the electrostatic potential using the CHELPG algorithm under the constraint that the sum of the partial charges should reproduce the total molecular charge. The thus obtained values were then averaged over all symmetry equivalent atoms in order to get a homogeneous distribution.

#### Molecular dynamics simulations

The dimensions of the simulations boxes were 5 × 5 × 45 nm, with 22.5 nm dedicated to each phase (aqueous and organic). In the case of polarisation by charge difference, the aqueous phase contained *p*_A_ positive ions (Li^+^) and *n*_A_ negative ions (Cl^−^) such that *p*_A_ + *n*_A_ = 120 and *p*_A_ − *n*_A_ = *c*, where *c* is the desired charge difference. This gives a LiCl concentration of ∼200 mM. Similarly, the organic phase contained *p*_O_ positive ions (BA^+^) and *n*_O_ negative ions (TB^−^) such that *p*_O_ + *n*_O_ = 12 and *p*_O_ − *n*_O_ = *c*. This gives a BATB concentration of ∼20 mM. The electrostatic interactions were calculated using PME summation. However, in order to avoid artefacts due to the three dimensional periodic boundary conditions, the simulations were periodic only the *x* and *y* directions. Consequently, a pseudo two-dimensional Ewald summation was used. Walls where therefore placed at *z* = 0 nm and *z* = 45 nm to contain the system. In the case of polarisation by a constant electric field, 3D periodic boundary conditions were used as well as 3D PME summation. The system energy was then minimized until a force threshold of 100 kJ mol^−1^ nm^−1^ was reached. Then, the box was quickly equilibrated for 1 ns in the NVT ensemble at 293 K using the “V-rescale” thermostat^46^ with a time constant coupling of 0.5 ps and a time step of 2 fs. Finally, the simulations were run in the NPT ensemble for 100 ns to 200 ns, with a time step of 2 fs, at 293 K and 1 bar using the “V-rescale” thermostat (time constant coupling of 0.5 ps) and Berendsen barostat^47^ (time constant coupling of 1 ps). The semi-isotropic pressure coupling was used with a compressibility of 0 bar^−1^ in the *x* and *y* directions and of 4.5 × 10^−5^ bar^−1^ in the *z* direction. The LINCS algorithm^48^ was used to constrain the bonds containing hydrogen atoms. The first 10 ns of each simulations where discarded before analysis.

#### Data analysis

The potential and ion density profiles were obtained with the standard tools of GROMACS 2018.1, *i.e.* “gmx_density” and “gmx_potential”. The permittivity was computed using the Kirkwood–Frölich formula:^15^
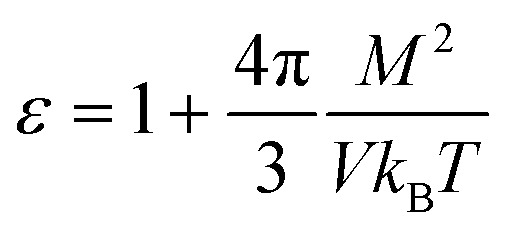
where *M*^2^ is the variance of the square of the total dipole moment in the volume *V*. The other symbols have their usual meaning. The total dipole moment was calculated using a homemade code based of the library “MDanalysis”.^49,50^ Briefly, the simulation box was divided in slices of 0.5 nm. In each slice, the dipoles of all the solvent molecules was averaged over the duration of the simulation. The variance of the square of this value was then used in the formula. This procedure gives a permittivity of the TIP4P water model in agreement with published values.^51^

### Second harmonic generation

SHG data were acquired with the setup presented in Fig. S4.† The pulses (30 ps, 50 Hz, 1000 nm) were generated by a parametric generator Ekspla PG400 series, pumped by a laser Ekspla PL2230 series. The beam was focused on the water–DCE interface from below, in total internal reflection, by a 100 mm lens (spot size ∼100 μm). The second harmonic of the probe beam (500 nm) was then collected by a 100 mm lens and sent to a Triax 320 spectrophotometer. The signal was then detected by a photomultiplier tube Hamamatsu R928, sent to a boxcar averager and recorded in a computer. The polarisation of the beam was made circular with the help of a quarter-wave plate. The typical pulse energies lied between 8 μJ and 10 μJ.

The potential-dependent SHG signal was recorded by polarising the cell with the help of a four-electrode potentiostat Metrohm Autolab PGSTAT 204. The traces were obtained by recording the SHG signal while cyclic voltammograms were performed in the cell. The scan rate of the voltammograms was 1 mV s^−1^ and the SHG signal was integrated over 1 s (50 pulses) per point during the scan. Five voltammetric cycles were averaged for each concentrations.

### Microhole-supported electrochemical impedance spectroscopy

The electrochemical impedance spectra were measured in a custom Teflon cell, made of two compartments separated by a 30 μM thick Kapton film (Fig. S5†). A conical hole with an entrance diameter of ∼35 μM and exit diameter of ∼23 μM was drilled in the film by laser ablation, prior to insertion in the cell. The larger hole was always located on the aqueous side of the cell. The interface was then polarised in a two-electrode configuration with the help of a Metrohm Autolab PGSTAT 204 potentiostat equipped with a FRA 32 frequency analyser. Each electrode was made with an AgCl coated silver wire. The cell was filled first with the aqueous solution, followed by the organic solution in order to form the ITIES on the organic side of the cell.^52^

The impedance spectra were recorded by applying sinusoidal perturbations of frequencies ranging from 30 kHz to 100 Hz and with a voltage amplitude of 20 mV. The interface was polarised for 30 s at the desired potential difference before the beginning of the impedance measurements. The potential window was scanned from negative to positive potentials by steps of 100 mV; each measurements succeeded immediately the previous one. The impedance spectra were analysed by fitting the data with the model presented in Fig. S6.†^53,54^ All experiments were carried out in a faradaic cage.

## Conflicts of interest

Authors declare no competing interests.

## Supplementary Material

SC-011-D0SC00685H-s001
