# Supplementary material for: How to polarise an interface with ions: the discrete Helmholtz model
Source: Chem Sci. 2020 May 18;11(39):10807–13. doi: 10.1039/d0sc00685h (PMC8162426; doi:10.1039/d0sc00685h)
Supplement: SC-011-D0SC00685H-s001 [file SC-011-D0SC00685H-s001.pdf]

Electronic Supplementary Material (ESI) for Chemical Science.
This journal is © The Royal Society of Chemistry 2020

Supplementary material for:

How to polarise an interface with ions: the discrete Helmholtz model.

Grégoire C. Gschwend, Astrid Olaya, Hubert H. Girault

This PDF file includes:

Figs. S1 to S10.

Tables S1 and S2.

Simulated charge densities:

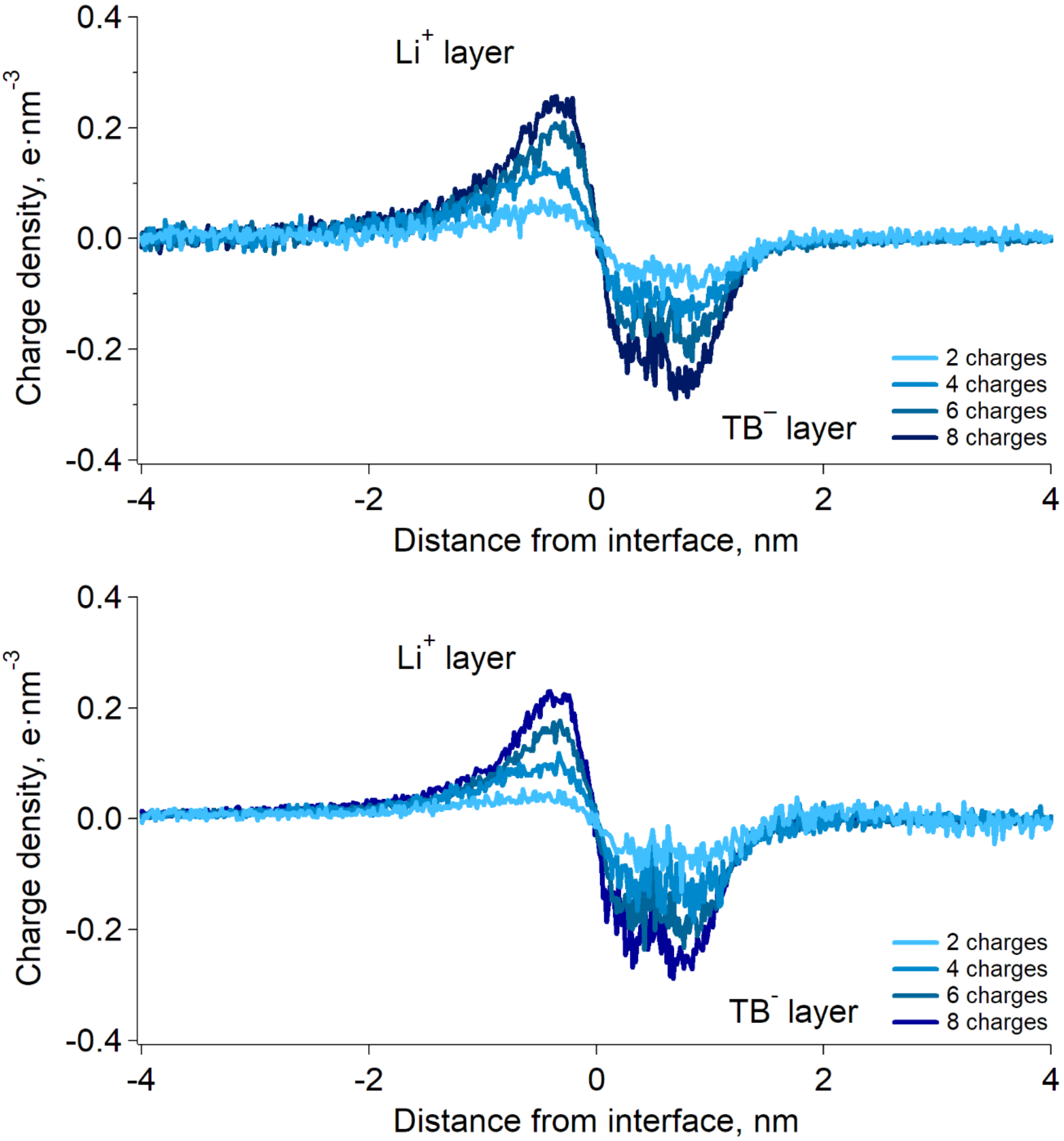

Figure S1: Charge density profiles simulated with the method of "charge difference". Top: 200 mM LiCl in water, 20 mM BATB in DCE. Bottom, 20 mM LiCl in water, 20 mM BATB in DCE.

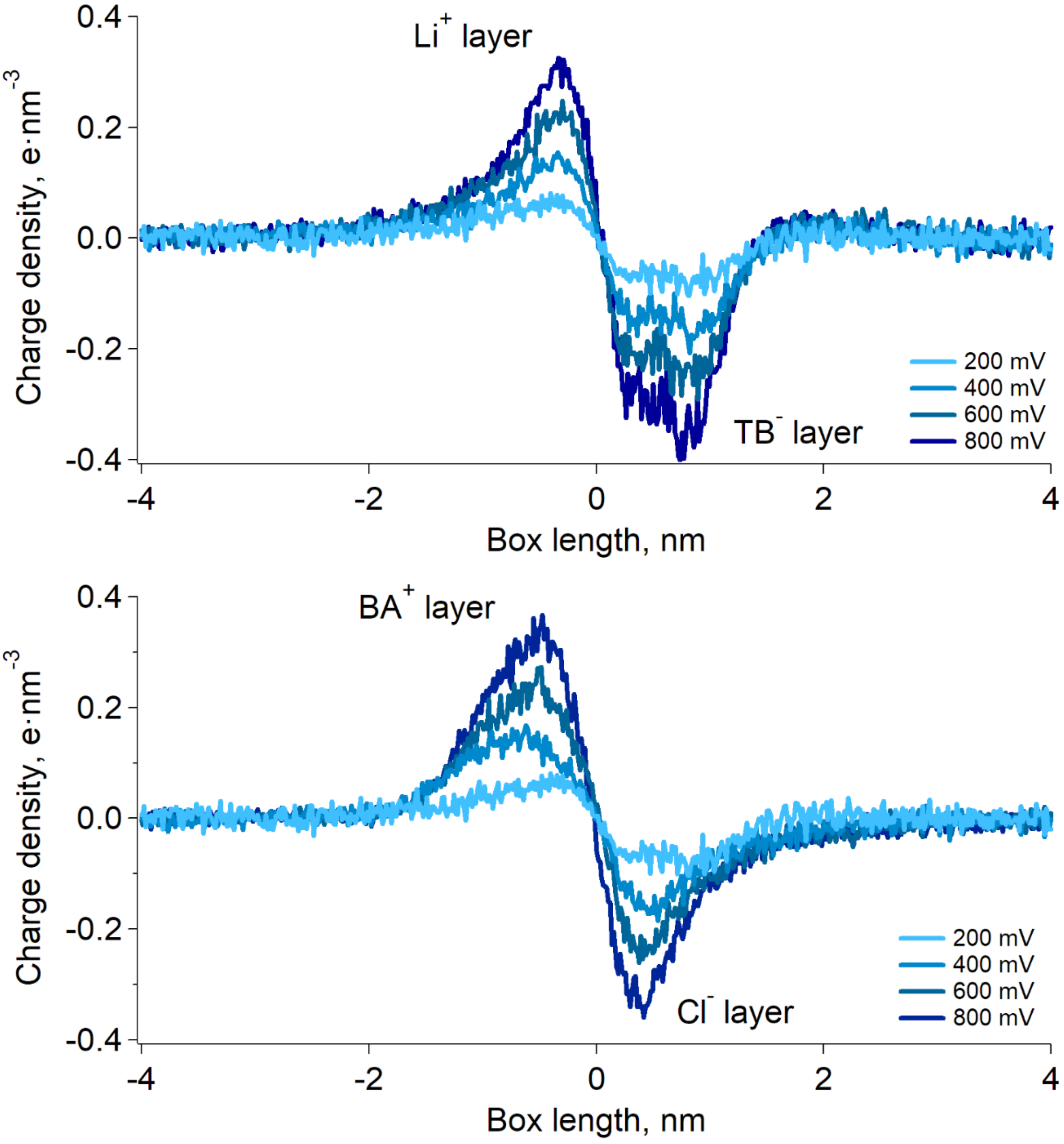

Figure S2: Charge density profiles simulated with the method of constant electric field, 200 mM LiCl in water, 100 mM BATB in DCE. Top: positive polarisations. Bottom: negative polarisations.

Simulated potential profiles:

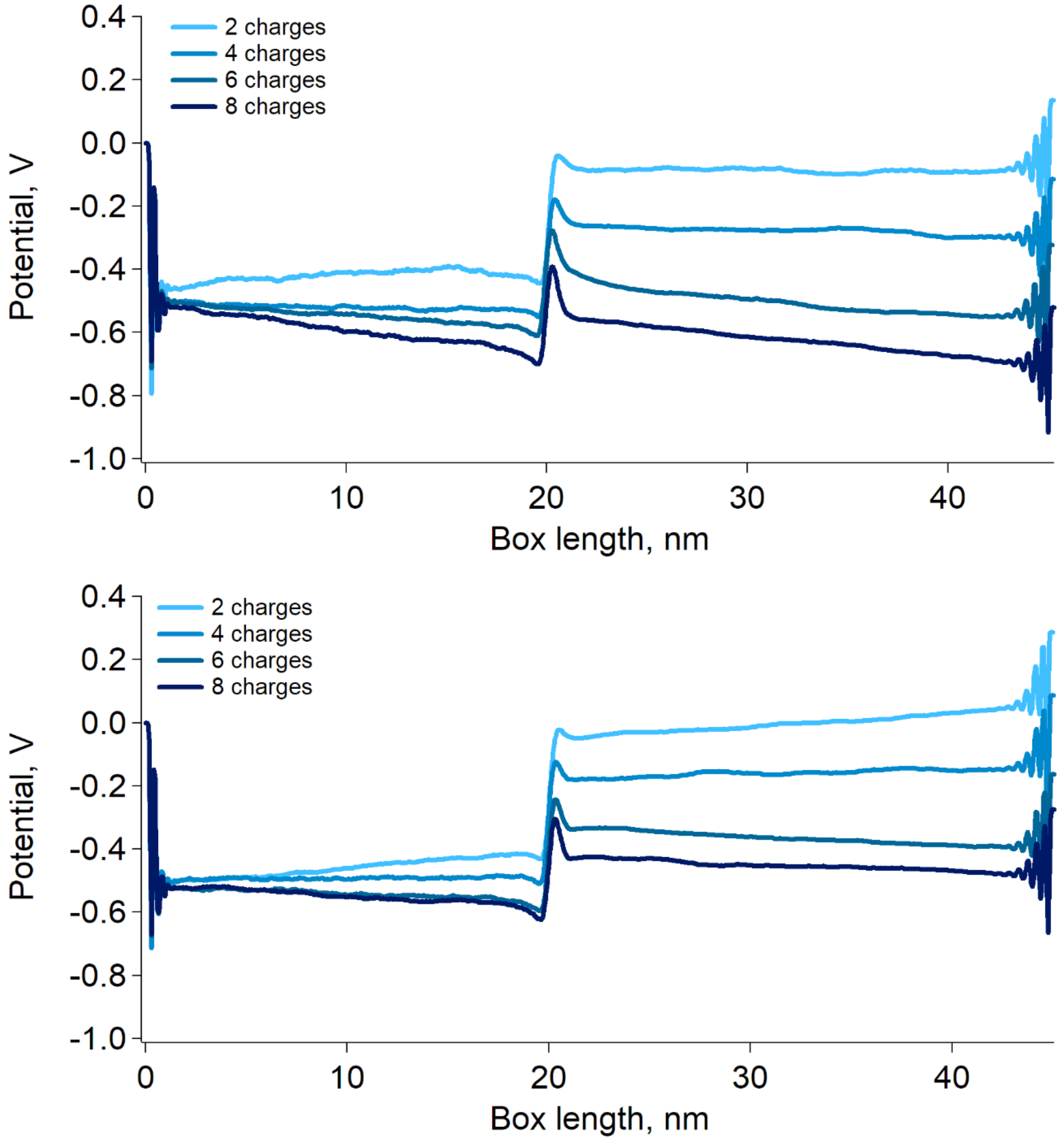

Figure S3: Simulated potential profiles simulated with the method of “charge difference”. Top: 200 mM LiCl in water, 100 mM BATB in DCE. Bottom: 200 mM LiCl in water, 20 mM BATB in DCE.

Second harmonic generation setup:

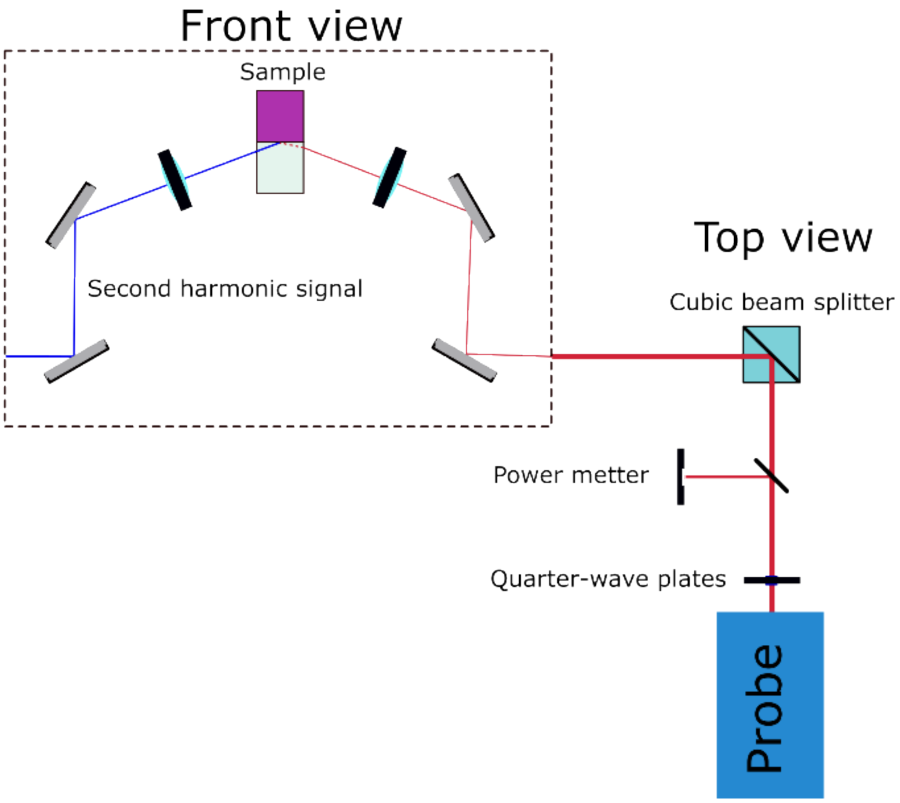

Figure S4. Schematic representation of the second harmonic generation setup.

Electrochemical impedance spectroscopy setup:

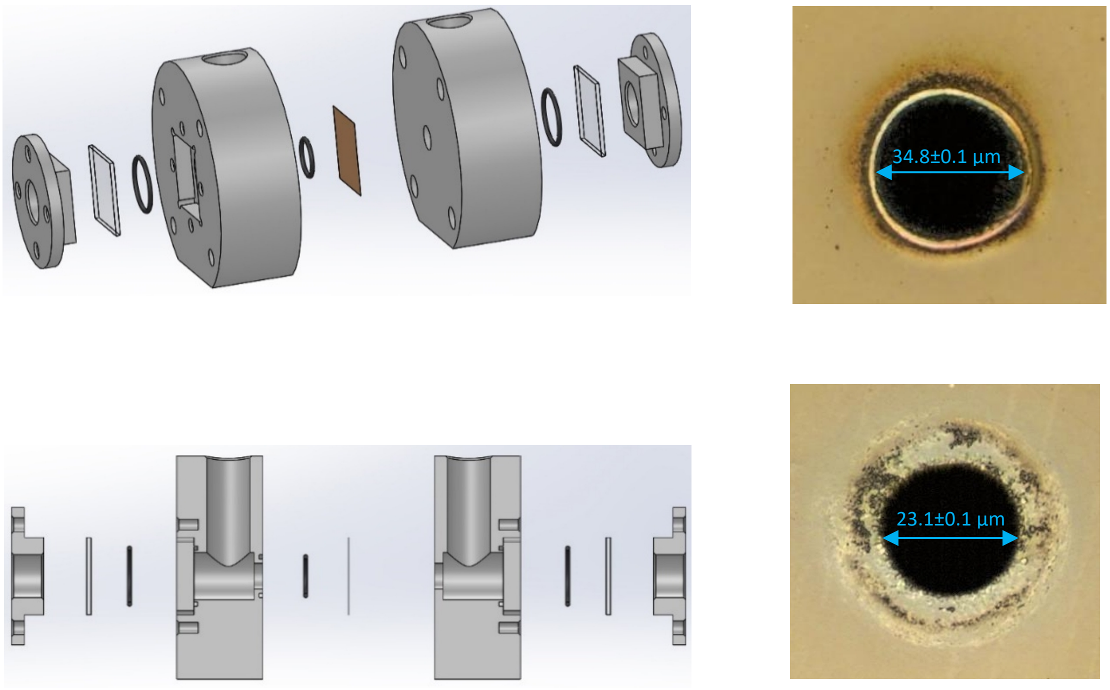

Figure S5. Left: schematic representation of the microhole electrochemical cell (general view and side view). Right: confocal microscopy view of the laser-ablated hole in the Kapton film used to support the micro liquid-liquid interface. The top hole was on the aqueous side of the cell while the bottom hole was on the organic side. The ITIES was formed on the organic side of the cell (*i.e.* with the bottom hole).

Equivalent circuit for impedance spectra fitting:

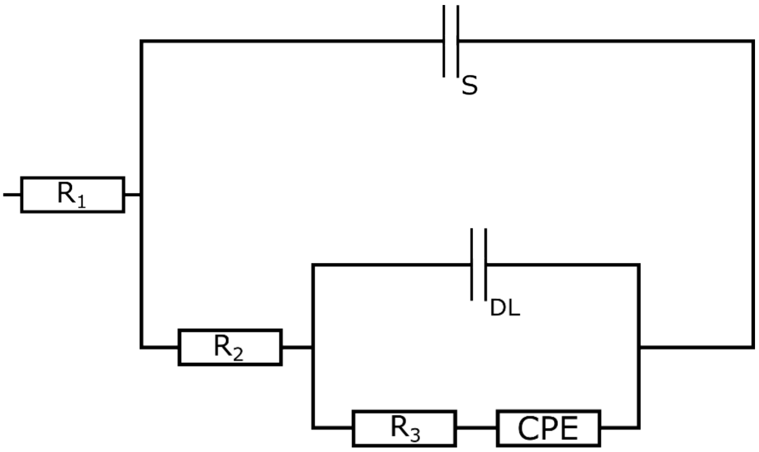

Figure S6. Equivalent circuit used to fit the electrochemical impedance data. R_1 is the bulk resistance, R_2 is the resistance inside the micro-pore, R_3 is the charge transfer resistance at the interface, CPE is a constant phase element used to simulate to non-ideal diffusion impedance at the interface, S is the stray capacitance of the cell and DL is the capacitance of the interface.

Electrochemical impedance spectroscopy fitting parameters:

[BATB]	R_1	C_S	R_2	C_{DL}	R_3	Y_0	N	X^2
1 mM	65743 (4)	5.31e-11 (6)	2.68e5(21)	2.63e-11 (12)	4.17e6 (5)	2.29e-9(7)	0.56919(2)	0.002661
	68495 (3)	5.71e-11 (5)	3.64e5 (22)	2.31e-11 (11)	5.22e6 (4)	7.48e-10 (8)	0.7088 (2)	0.004173
	67164 (4)	5.57e-11 (6)	3.29e5 (22)	2.42e-11 (11)	5.15e6 (3)	6.99e-10 (8)	0.71529 (2)	0.004013
	67982 (4)	5.66e-11 (6)	3.56e5 (24)	2.31e-11 (12)	5.50e6 (4)	4.26e-10 (8)	0.75953 (2)	0.004787
	67014 (5)	5.55e-11 (7)	3.30e5 (29)	2.38e-11 (15)	5.47e6 (5)	2.83e-10 (9)	0.78279 (2)	0.006382
	68211 (3)	5.72e-11 (4)	3.74e5 (18)	2.26e-11 (9)	5.67e6 (3)	2.94e-10 (6)	0.80028 (1)	0.002843
	68740 (3)	5.80e-11 (4)	4.07e5 (18)	2.20e-11 (9)	5.59e6 (3)	4.98e-10 (7)	0.76318 (1)	0.003264
	68308 (3)	5.74e-11 (4)	3.90e5 (18)	2.24e-11 (9)	5.38e6 (3)	7.23e-10 (7)	0.72841 (2)	0.003196
	66449 (3)	5.46e-11 (5)	3.14e5 (17)	2.44e-11 (9)	4.84e6 (2)	1.31e-9 (6)	0.6593 (1)	0.002096
10 mM	22726 (4)	6.23e-11 (1)	8.53e5 (11)	1.97e-11 (5)	1.94e6 (4)	2.77e-9 (5)	0.60239 (1)	0.002410
	23283 (3)	6.30e-11 (1)	9.52e5 (9)	2.18e-11 (4)	2.33e6 (3)	5.18e-10 (4)	0.78761 (1)	0.002441
	22814 (4)	6.25e-11 (1)	8.81e5 (11)	2.07e-11 (5)	2.36e6 (4)	3.39e-10 (5)	0.8133 (1)	0.003204
	21922 (6)	6.13e-11 (2)	7.24e5 (20)	1.84e-11 (5)	2.20e6 (5)	2.47e-10 (5)	0.81663 (1)	0.004149
	23037 (5)	6.27e-11 (2)	9.21e5 (14)	2.11e-11 (6)	2.52e6 (5)	2.17e-10 (6)	0.84577 (1)	0.005082
	22876 (4)	6.26e-11 (1)	9.02e5 (11)	2.12 e-11 (5)	2.33e6 (3)	3.87e-10 (5)	0.80947 (1)	0.003306
	23898 (4)	6.36e-11 (1)	1.07e6 (10)	2.41e-11 (6)	2.42e6 (4)	4.50e-10 (6)	0.81361 (1)	0.004929
	22977 (3)	6.26e-11 (1)	9.13e5 (8)	2.16e-11 (4)	2.18e6 (2)	8.19e-10 (4)	0.75175 (1)	0.001720
	22559 (3)	6.19e-11 (1)	8.20e5 (10)	2.00e-11 (4)	1.98e6 (3)	1.70e-9 (4)	0.67153 (1)	0.001873
50 mM	29894 (2)	6.25e-11 (1)	8.47e5 (5)	2.93e-11 (4)	1.49e6 (2)	1.35e-9 (3)	0.73764 (1)	0.001307
	30442 (2)	6.31e-11 (1)	9.20e5 (6)	3.23e-11 (5)	1.76e6 (3)	2.97e-10 (4)	0.88103 (1)	0.002954
	29922 (3)	6.24e-11 (1)	8.40e5 (8)	2.84e-11 (6)	1.73e6 (3)	2.37e10 (4)	0.87679 (1)	0.003151
	28923 (3)	6.15e-11 (1)	7.37e5 (10)	2.55e-11 (6)	1.65e6 (3)	2.27e-10 (4)	0.86603 (1)	0.002840
	29228 (3)	6.18e-11 (1)	7.73e5 (8)	2.68e-11 (5)	1.67e6 (3)	2.46e-10 (4)	0.87198 (1)	0.002469
	30012 (3)	6.25e-11 (1)	8.57e5 (7)	2.98e-11 (5)	1.68e6 (3)	2.85e-10 (4)	0.87838 (1)	0.002859
	30100 (2)	6.26e-11 (1)	8.71e5 (6)	3.07e-11 (5)	1.63e6 (3)	3.61e-10 (4)	0.86893 (1)	0.002735
	29783 (2)	6.23e-11 (1)	8.33e5 (6)	2.97e-11 (5)	1.53e6 (2)	6.07e-10 (4)	0.82542 (1)	0.002365
	29067 (3)	6.13e-11 (1)	7.26e5 (8)	2.57e-11 (5)	1.31e6 (3)	2.52e-9 (4)	0.67219 (1)	0.001832

Table S1: Fitting parameters of the electrochemical impedance spectroscopy experiment at the microhole supported ITIES. LiCl 10 mM in the aqueous phase, various concentrations of BATB (see table) in the DCE phase.

[BATCPB]	R_1	C_S	R_2	C_{DL}	R_3	Y_0	N	X^2
1 mM	64275 (3)	5.29e-11 (3)	5.00e5 (16)	2.02e-11 (6)	3.74e6 (15)	3.24e-9 (9)	0.46666 (4)	0.003243
	66346 (3)	5.50e-11 (2)	6.35e5 (14)	1.98e-11 (5)	5.97e6 (7)	1.14e-9 (11)	0.61688 (3)	0.004707
	67406 (3)	5.59e-11 (2)	7.11e5 (14)	1.95e-11 (5)	6.98e6 (6)	6.23e-10 (11)	0.69491 (3)	0.005263
	67310 (2)	5.57e-11 (2)	6.90e5 (13)	1.93e-11 (4)	7.04e6 (5)	3.81e-10 (10)	0.73595 (2)	0.004113
	65674 (4)	5.39e-11 (4)	5.50e5 (23)	1.94e-11 (8)	5.74e6 (11)	2.48e-10 (11)	0.74015 (2)	0.006853
	65709 (3)	5.45e-11 (3)	5.89e5 (17)	1.96e-11 (6)	6.03e6 (8)	5.26e-10 (10)	0.68259 (2)	0.005083
	65453 (3)	5.43e-11 (3)	5.73e5 (15)	1.95e-11 (5)	5.56e6 (7)	1.20e-9 (9)	0.59548 (3)	0.003638
	65627 (3)	5.46e-11 (3)	5.90e5 (16)	1.93e-11 (6)	5.44e6 (9)	1.93e-9 (11)	0.54289	0.004186
	67088 (2)	5.59e-11 (2)	6.92e5 (14)	1.88e-11 (5)	6.30e6 (8)	2.10e-9 (12)	0.55008 (4)	0.004313
10 mM	43177 (3)	5.45e-11 (4)	3.25e5 (24)	1.34e-11 (10)	-1.02e6 (41)	2.91e-8 (3)	0.24021 (5)	0.000636
	47205 (1)	6.08e-11 (1)	8.49e5 (5)	2.12e-11 (2)	2.17e6 (1)	1.13e-9 (2)	0.70138 (0)	0.000491
	47289 (1)	6.07e-11 (1)	8.54e5 (5)	2.11e-11 (2)	2.27e6 (1)	6.87e-10 (2)	0.74867 (0)	0.000548
	46565 (1)	6.00e-11 (1)	7.67e5 (5)	2.00e-11 (2)	2.26e6 (1)	5.33e-10 (2)	0.76335 (0)	0.000454
	45766 (2)	5.88e-11 (1)	6.44e5 (10)	1.83e-11 (2)	2.04e6 (2)	4.34e-10 (2)	0.75784 (0)	0.000785
	46206 (1)	5.93e-11 (1)	6.99e5 (7)	1.87e-11 (2)	2.15e6 (2)	3.70e-10 (2)	0.77232 (0)	0.000469
	46935 (1)	6.04e-11 (1)	8.30e5 (5)	2.07e-11 (2)	2.35e6 (1)	5.40e-10 (2)	0.76500 (0)	0.000524
	47210 (1)	6.09E-11 (1)	8.89E5 (6)	2.18E-11 (2)	2.32E6 (2)	7.74E-10 (3)	0.74511 (1)	0.000923
	47434 (1)	6.10e-11 (1)	9.02e5 (5)	2.23e-11 (3)	2.18e6 (2)	1.25e-9 (3)	0.69868 (1)	0.000898
50 mM	31647 (2)	6.50e-11 (1)	9.30e5 (6)	2.85e-11 (6)	1.12e6 (4)	2.33e-8 (6)	0.4453 (3)	0.001386
	30879 (2)	6.38e-11 (1)	7.94e5 (8)	2.42e-11 (5)	1.46e6 (3)	8.85e-10 (3)	0.76681 (1)	0.001638
	30646 (2)	6.35e-11 (1)	7.58e5 (8)	2.32e-11 (5)	1.52e6 (3)	5.26e-10 (3)	0.79975 (0)	0.001294
	30002 (3)	6.25e-11 (2)	6.43e5 (15)	2.03e-11 (5)	1.46e6 (4)	4.77e-10 (3)	0.78767 (0)	0.001850
	30073 (3)	6.26e-11 (2)	6.53e5 (13)	2.04e-11 (5)	1.49e6 (3)	4.47e-10 (3)	0.79309 (0)	0.001462
	31119 (2)	6.40e-11 (1)	8.14e5 (6)	2.46e-11 (4)	1.61e6 (2)	4.35e-10 (2)	0.82560 (0)	0.001093
	31268 (1)	6.41e-11 (1)	8.35e5 (5)	2.57e-11 (3)	1.60e6 (2)	5.37e-10 (2)	0.81817 (0)	0.000887
	31386 (2)	6.43e-11 (1)	8.53e5 (5)	2.63e-11 (4)	1.56e6 (2)	6.45e-10 (2)	0.80678 (0)	0.000972
	31038 (2)	6.38e-11 (1)	7.89e5(7)	2.44e-11 (5)	1.39e6 (3)	1.57e-9 (3)	0.71812 (1)	0.001243

Table S2: Fitting parameters of the electrochemical impedance spectroscopy experiment at the microhole

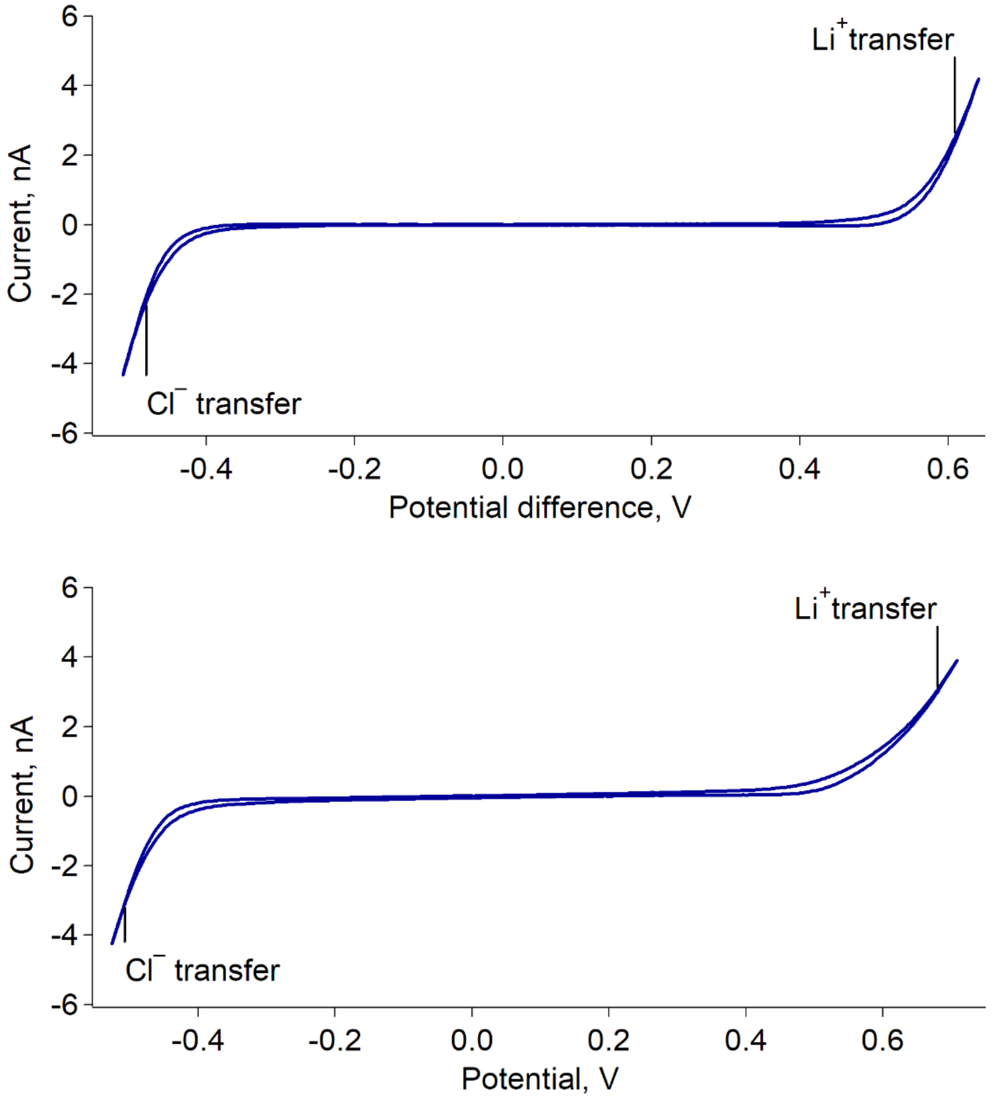

supported ITIES. LiCl 10 mM in the aqueous phase, various concentrations of BATCPB (see table) in the DCE phase.

<u>Cyclic voltammograms and impedance spectra at the microhole supported ITIES</u>:

Figure S7: Cyclic voltammograms at the microhole supported liquid-liquid interface. Top: LiCl 10 mM in water, BATB 1mM in DCE. Bottom: LiCl 10mM in water, BATCPB 1 mM in DCE.

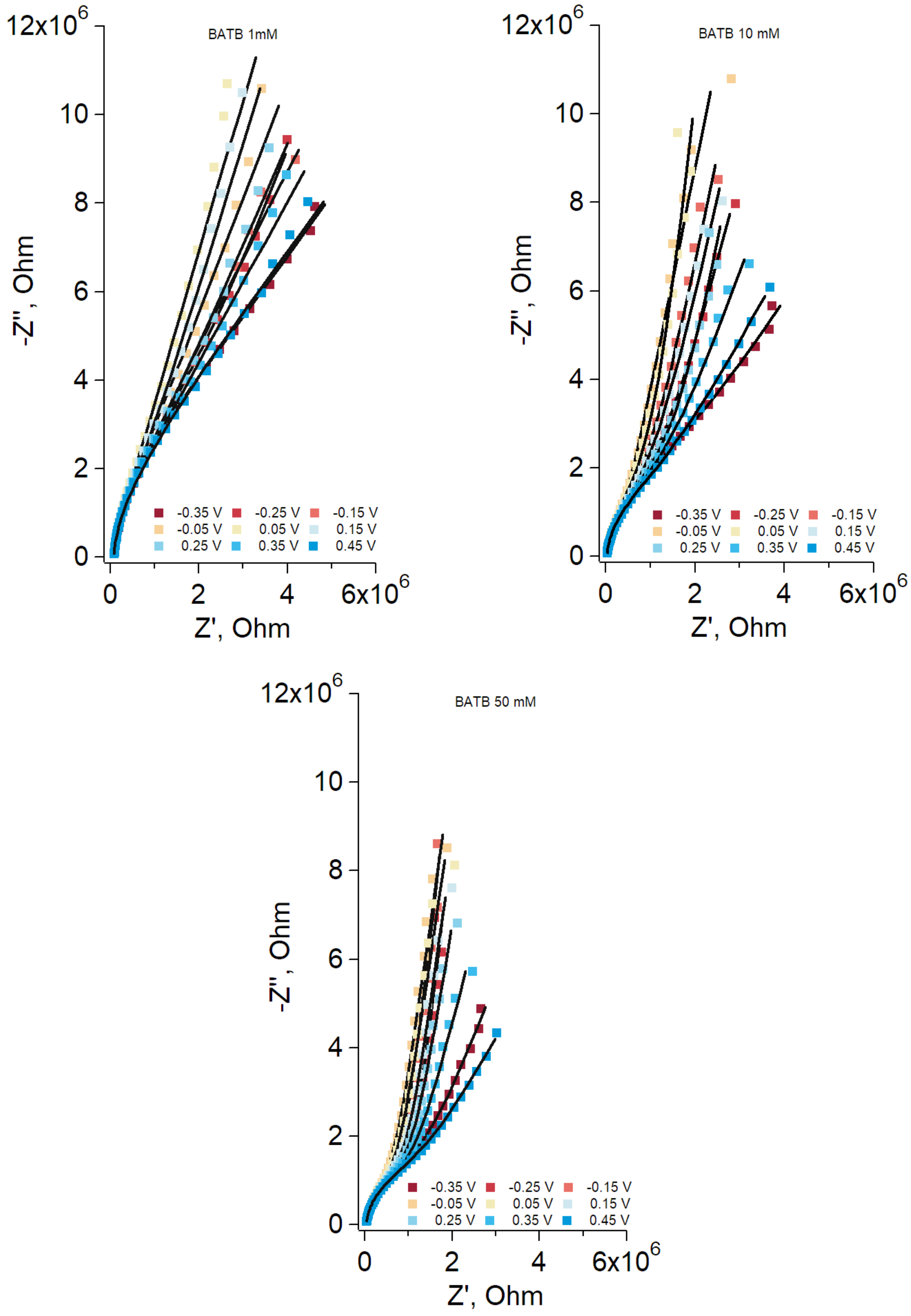

Figure S8: Nyquist plots of the electrochemical impedance spectroscopy experiments at the micro ITIES. A) LiCl 10 mM, BATB 1 mM, B) LiCl 10 mM, BATB 10 mM, C) LiCl 10 mM, BATB 50 mM.

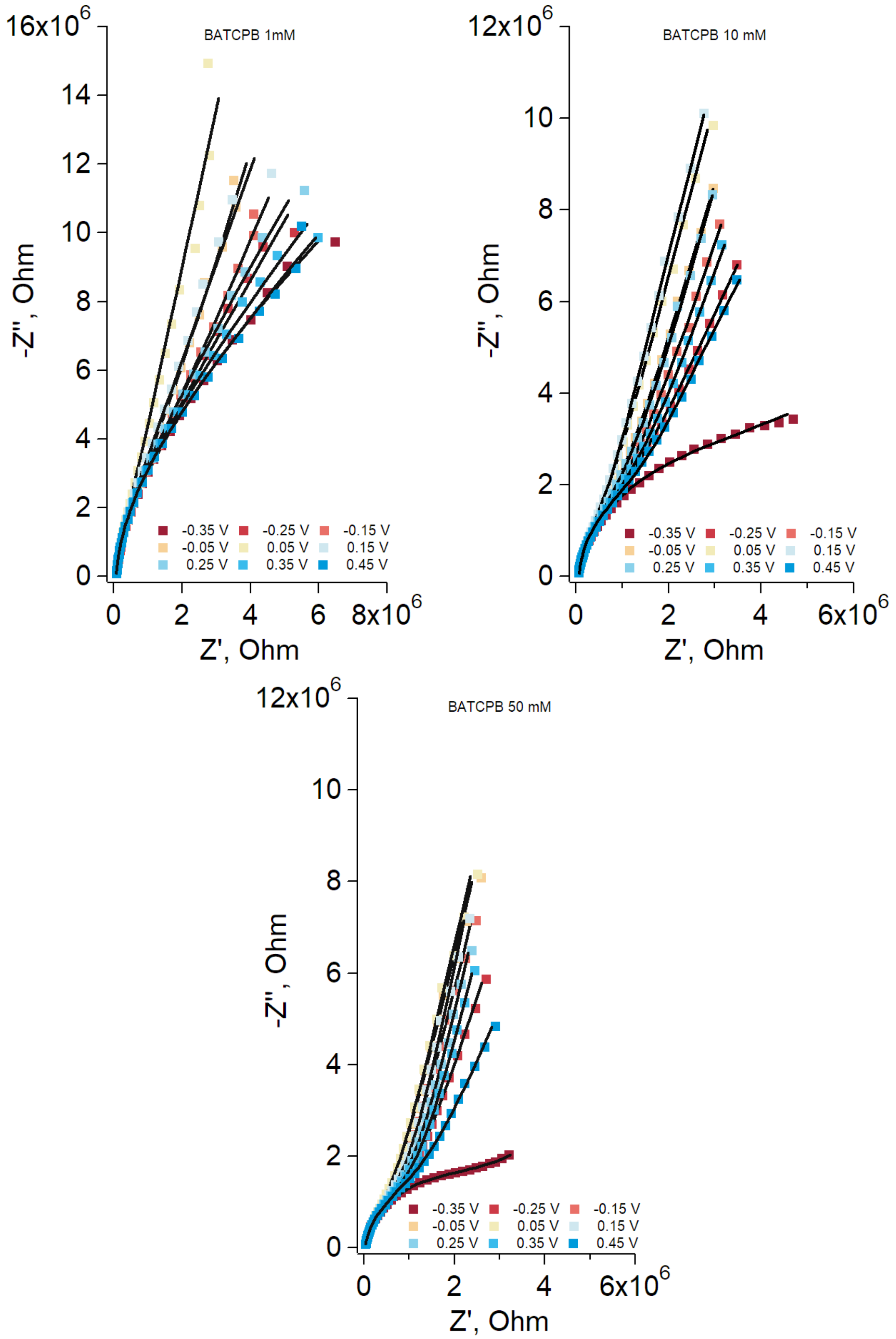

Figure S9: Nyquist plots of the electrochemical impedance spectroscopy experiments at the micro ITIES A) LiCl 10 mM, BATCPB 1 mM, B) LiCl 10 mM, BATCPB 10 mM, C) LiCl 10 mM, BATCPB 50 mM.

Capacitance of the LiCl/BATCPB micro interface at various electrolyte concentrations:

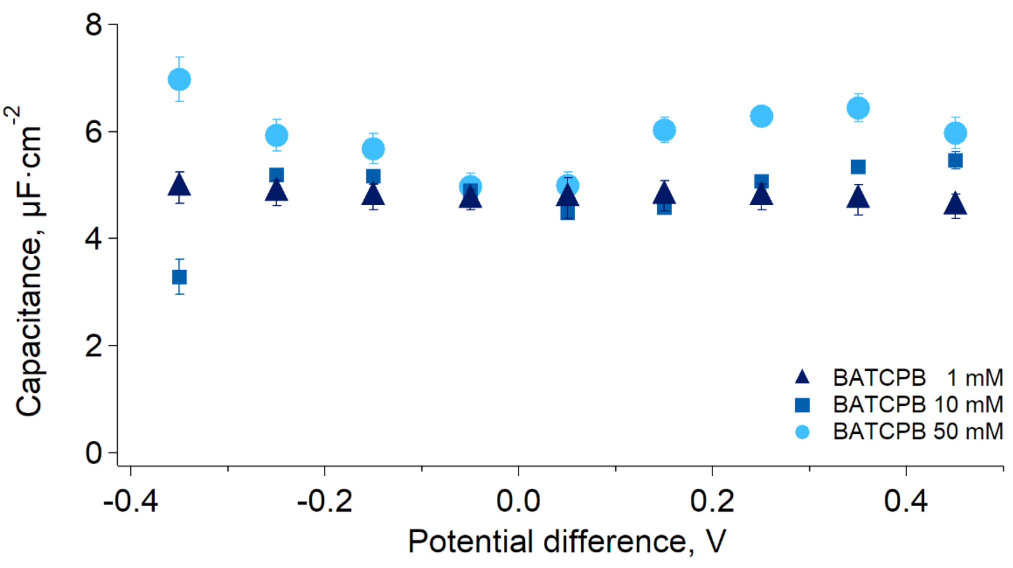

Figure S10: Capacitance of the micro ITIES formed between an aqueous solution of LiCl 10mM and BATPB at various concentrations in DCE.
